# Beyond “male eating disorders”: the risks of reinforcing gendered frames in eating disorder discourse

**DOI:** 10.1007/s40519-026-01918-5

**Published:** 2026-10-28

**Authors:** James Downs

**Affiliations:** Peer Researcher with Lived Experience, Cardiff, Wales UK

Dear Editor-in-Chief,

There is growing recognition that eating disorders in men have long been under-acknowledged, under-researched, and under-treated. This shift in awareness is both necessary and overdue. At the same time, I am concerned that current attempts to improve visibility for men within eating disorder discourse may carry limitations and risks of their own, particularly when they leave intact the assumptions that produced the original disparity. One reductive gendered narrative may simply be supplemented with another, without the gendered frame itself being interrogated.

Historically, eating disorders have been shaped, clinically and culturally, by assumptions about femininity. They have often been imagined through a narrow visual and psychological script: the young woman with a fear of fatness and a frail or visibly underweight body, whose pathology is read as a need for control or the pursuit of appearance ideals. Against this backdrop, recent work on muscularity-oriented concerns, compulsive exercise, and a drive for leanness has corrected clinically important omissions in models centred on thinness [1]. These patterns are undoubtedly relevant and must not be ignored. However, difficulty arises when they function as defining features of “male eating disorders”, because this risks reifying a new set of gendered archetypes in which male distress becomes legible only when it conforms to a pre-determined masculine form.

From my own perspective as a male with lived experience of anorexia and bulimia, I have observed the construction of “male eating disorders” over the course of my longstanding illness. When I was first diagnosed with anorexia, more than twenty years ago, people’s responses were often dismissive or disbelieving. Professionals openly doubted why I “wanted” to have a “female” disorder, and often treated me as a rare case that was extremely unusual to see in clinical practice. Disbelief has become less common over time, and the possibility that men may have eating disorders is now widely accepted. This represents real progress. I have found, however, that a lack of awareness has not necessarily been replaced with understanding, but with a different set of assumptions.

These days, my experiences are still often interpreted through narratives that do not fit: assumptions that a man with an eating disorder must primarily be preoccupied with muscularity, masculinity, or leanness in order to be stereotypically attractive. It may also be assumed that because I am gay, I want to lose weight to become more feminine, or that my difficulties are somehow rooted in a refusal to accept my sexuality. These assumptions may be subtler than outright scepticism and ignorance, but they remain reductive and limiting nonetheless. For those who do not recognise themselves in these emerging typologies, the effect may be one of continued invisibility, or visibility only on distorted terms. My concern is not simply whether men are included in eating disorder discourse, but to ensure that a diversity of male experiences are rendered intelligible once they are.

My point is not that gender is irrelevant. It plainly matters. Gender norms shape bodily experiences, ideals, visibility, and the social meanings attached to food, control, thinness, strength, and vulnerability. Yet gender neither acts alone nor uniformly. Eating disorders unfold through intersections with sexuality, age, race, class, disability, neurodivergence, trauma, culture, relational context, and differences in severity, duration, and symptom pattern. The distinction I wish to draw is between essentialist typologies, which treat gender as predictive, and gender-informed approaches, which examine how gender interacts with a person’s unique circumstances. One response to the very real complexity of eating disorders is to keep adding categories and subgroupings. Yet this additive logic leaves the classificatory frame intact. I would instead challenge the assumption that categories can capture in advance what distress will mean or how it will manifest. A more adequate framework would respond to people as individually situated and context-bound, rather than asking identity categories to do the interpretive work for us [2].

Existing scholarship points in this direction. Recent work on eating disorders in boys and men has emphasised not a single male presentation, but the diverse and uneven realities through which such difficulties are lived and responded to [3]. Rather than treating masculinity as a stable explanatory category, this literature points to a plurality of masculinities, shaped in different ways by sexuality, age, race, class, culture, trauma, neurodivergence, embodiment, and the highly personal functions that food, exercise, and body management can come to serve in individual lives. It also shows how lived experience accounts frequently exceed the narrow and often ill-fitting categories through which eating disorders are clinically recognised. Read in this light, male experiences of eating disorders are more heterogeneous, shifting, and context-bound than gendered typologies can comfortably contain.

For these reasons, I would suggest that the task is not to refine gendered categories indefinitely, but to loosen their grip. Rather than asking how eating disorders differ in men and women as if these were internally coherent groups, it may be more useful to ask how particular sociocultural pressures, relational histories, embodied vulnerabilities, and identity formations converge in singular lives. This would not mean abandoning attention to gender, but refusing to let it dictate the terms of understanding in advance. It would also mean resisting the tendency to treat inclusion as a matter of representation alone, as though justice were achieved once each demographic group had its corresponding diagnostic narrative.

The increased attention to eating disorders in men therefore presents an important opportunity, but also a risk. It can either extend the life of binary frameworks by furnishing them with updated content, or it can prompt a more searching reappraisal of the assumptions that have organised the field of eating disorders for so long. I would argue for the latter.

Yours faithfully,

James Downs

## Data Availability

No datasets were generated or analysed during the current study.
